# 

**Published:** 1894-12

**Authors:** 


### Mississippi Valley Dental Society.

The annual meeting of this, the oldest dental society in this country if not in the world, will be held in Cincinnati, March next.

The Executive Committee is at work to make it an occasion of great interest; and special effort will be made to have in attendance the largest possible number of the members of twenty-five years ago and before.

A historical sketch, especially of its earlier existence, will be given, with the object of presenting the steps of progress in the interest of the profession, which it has been instrumental in inaugurating and promoting. The labors of the early workers in the profession should not be forgotten; they will not, of course, in the minds of those who were participants; but those who have come on to the stage in recent years can only know of these things by written or verbal accounts.

It is earnestly to be hoped that there will be a large number present on that occasion; not only of the older members but the younger members, and, indeed, of those who have not been members as well; that all together may participate in the work and enjoyment of the occasion.

The precise date will be given in the programme in the near future.

### DIRECTORY OF DENTAL SOCIETIES.

*American Dental Association*—Meets first Tuesday in August, 1894, at Old Point Comfort. Pres., J. D. Patterson, Kansas City, Mo.; Rec. Sec., Geo. H. Cushing, Chicago.

*Southern Dental Association*—Will meet in 1894. At Old Point Comfort. Pres., B. Holly Smith, Baltimore, Md.; Secretary, D. R. Stubblefield, Nashville, Tenn.

### STATE DENTAL SOCIETIES.

*Alabama Dental Association*—Meets annually. Next meeting at Montgomery, on the second Tuesday of April, 1894. Pres. T. M. Allen, Birmingham; Sec. S. W. Foster, Decatur.

*California State Dental Association*—Next meeting will be held in San Francisco, July, 1894. Pres., L. A. Teague, San Francisco; Rec. Sec., W. Z. King, San Francisco; Cor. Sec., C. E. Post, San Francisco.

*Colorado State Dental Society*—Meets 1st Tuesday in June, 1894, at Glenwood Springs. Pres. P. T. Smith, Denver; Sec., Sarah May Townsend, Denver.

*Connecticut State Dental Society*—Will hold a Union Meeting in connection with the Connecticut Valley Dental Society at Hartford, third Tuesday in May, 1894. Pres. C. C. Barker, Meriden; Rec. Sec. Geo. L. Parmele, Hartford.

*Dental Society of the State of Maryland and District of Columbia*—Meets annually. Next meeting at Washington City, on the second Tuesday in October, 1893 Pres. B. F. Coy, of Baltimore: Sec. M. W. Foster, Baltimore.

*Florida State Dental Association*—Meets annually. Next meeting at Talahassee, 1st Tuesday in May, 1894. Pres, F. E. Buck, Jacksonville; Cor. Sec. E. M. Sanderson, Jacksonville

*Georgia State Dental Society*—Meets in June, 1894, at Tybee Island; Pres. N. A. Williams, Valdosta; Rec. Sec. S. H. McKee, Americus; Cor. Sec. O. H. Mc Donald, Griffin.

*Illinois State Dental Society*—The next meeting will be held at Springfield, Ill., the second Tuesday in May, 1894. Pres. Garrett Newkirk, Chicago; Sec. Louis Ottofy, Chicago.

*Indiana State Dental Society*—Will meet at Lake Maxinkuckee, on the last Tuesday of June, 1894. Pres. W. M. Hindman, Vincennes; Secretary, G. E. Hunt Indianapolis.

*Iowa State Dental Society*—Meets annually. Next meeting in Council Bluffs, on the second Tuesday in May, 1894. Pres. S. E. Hatch, Sioux City; Rec. Sec. G. W. Miller, Des Moines.

*Kentucky State Dental Association*—Meets annually. Next meeting in Louisville, Ky., 3rd Tuesday in June, 1894. Pres., J. H. Letcher, Danville; Sec., J. H. Baldwin, Louisville.

*Kansas State Dental Association*—Pres. C. E. Esterly, Laurence; Sec., J. P. Root, Kansas City. Next meeting will be held at Topeka, last Tuesday in April, 1894.

*Maine Dental Society*—Will meet on the third Tuesday in July, 1894, in Rockland. Pres. H. A. Kelley, Portland; Sec. C. E. Bryant, Pittsfield.

*Maryland State Dental Association*—Next annual meeting will be held (place not definitely known yet,) in June, 1894. Pres., B. Holly Smith, Baltimore; Rec. Sec, W. W. Dunbracco, Baltimore; Cor. Sec., R. C. Bradshaw, Baltimore.

*Massachusetts Dental Society*—Meets annually in Boston, in June. Pres. Josiah W. Ball, Boston; Rec. Sec., E. O. Kinsman, Cambridge.

*Michigan State Dental Association*—Meets annually. Next meeting at Ann Arbor, Tuesday, June 7th, 1894. Pres., N. S. Hoff: Ann Arbor; Sec., J. Ward House, Grand Rapids. Treas. Geo. H. Mosher, Jackson.

*Mississippi State Dental Association*—Next meeting the first Tuesday in May, 1894, at Natchez, Miss. Pres. Jas. B. Askew, Vicksburg; Cor. Sec. J. D. Killian, Greenville.

*Minnesota State Society*—Pres. C. H. Stearns, Zumbrota; Sec., C. W. Jones, St. Paul. Will meet in Minneapolis, first week, in September, 1894.

*Missouri Dental Association*—Meets at Excelsior Springs, Mo., second Tuesday in July, 1894. Pres., W. E. Tucker, Springfield, Mo.; Sec., Wm. Conrad, St. Louis.

*Nebraska State Dental Society*—Meets annually. Next meeting third Tuesday in May 1893, at Beatrice; Pres. J. J. Willey, Wahoo; Sec. C. R. Tefft, Lincoln.

*New Jersey State Dental Society*—Meets annually. The next meeting will be held at Asbury Park, onthe 18th, 19th, 20th, of July, 1894. Pres. E. M. Beesley, Belvidere; Sec. C. A. Meeker, Newark.

*New Hampshire Dental Society*—Meets Annually. Next meeting will be held in Concord, N. H.; time to be decided later. Pres. W. R. Blackstone, Manchester; Sec. Burton C. Russell, Keene.

*North Carolina State Dental Society*—Meets in Durham, on the first Tuesday in May, 1894. Pres. C. A. Rominger, Reidsville; Sec., J. E. Wyche, Oxford.

*North Dakota State Dental Society*—The next meeting will be held in Grand Forks, in 1894, the exact time will be given later. Pres. B. D. McLain, of Jamestown; Sec. R. B. Foster, Grand Forks.

*Ohio State Dental Society*—Meets annually. Next meeting at Columbus, first Tuesday of December, 1894. Pres. Chas. Welch, Wilmington; Sec. L. P. Bethel, Kent.

*Pennsylvania State Dental Society*—Will hold its next annual meeting at Cresson, Pennsylvania, July 10th, 1894. Pres., F. L. Bassett, Philadelphia; Cor. Sec., H. E. Roberts, Philadelphia.

*Rhode Island Dental Association*—Pres., S. W. Clark, Providence; Sec., H. W. Gillett, Newport, Meets quarterly, second Tuesday in July, October, January and April. Annual Meeting second Tuesday in July, at Providence.

*South Carolina State Dental Association*—Meets annually. Pres. C. S. Patrick, Charleston; Cor. Sec. H. J. Ray, Aiken. Next meeting at Columbia, S. C., in August, 1893.

*Tennessee Dental Association*—Meets annually. Next meeting at Nashville, March 26-29, 1894. Pres. W. J. Morrison, Nashville; Rec. Sec. W. W. Jones, Murfreesboro; Cor. Sec. S. B. Cook, Chattanooga.

*The Dental Society of the State of New York*—Meets annually on the second Wednesday in May. Next session at Albany, 9th and 10th of May, 1894. Pres. F. T. Van Woert, Brooklyn; Sec. C. S. Butler, Buffalo; Cor. Sec. R. Ottolengui, New York.

*Vermont State Dental Society*—Meets annually. Next meeting at White River Junction, Vt., third Wednesday in March, 1894. Pres. A. J. Parker, Bellows Falls; Sec. Thos. Mound, Rutland.

*Virginia State Dental Association*—Will meet at Old Point Comfort, in 1894, Exact time to be decided later. Pres. H. W. Campbell, Suffolk; Sec. J. Hall Moore, Richmond.

*Washington State Dental Society*—Meets Annually. Next meeting will be held at Tacoma, in the early part of May, 1894. Pres. P. H. Carlyon, Olympia; Sec., A. S. Oliver, Olympia.

*Wisconsin State Dental Society*—Meets annually. Next meeting at will be held at Janesville, Wis., the second Tuesday in July, 1894. Pres., W. C. Wendel, Milwaukee; Sec., C. A. Southwell, Milwaukee.

*West Virginia State Society.*—The annual meeting will be held at Parkersburg, on the first Wednesday of October, 1894. Pres. J. N. Mahan, Charleston; Sec. Geo. I. Keener, Morgantown.

### LOCAL SOCIETIES.

*American Academy of Dental Science*—Meets annually in Boston, Mass., on the first Wednesday in October. Pres. J. H. Batchelder; Rec. Sec. E. E. Hopkins; Cor. Sec. E. B. Hitchcock, Boston.

*Buffalo Dental Association*—Meets last Monday evening of each month. Annual meeting in June. Pres. F. H. Boswell; Sec. J. A. Stackhouse.

*Brooklyn Dental Society and Second District Society United*—Meets quarterly—Pres. T. H. Holly; Rec. Sec. A. N. Russell, Brooklyn.

*Central Dental Association of Northern New Jersey*—Meets bi-monthly; and annually. The next annual meeting will be held about the middle of February, 1894. Pres. Harvey Iredell, New Brunswick; Sec. Wm. L. Fish, Newark.

*Chicago Dental Society*—Meets monthly. Pres. J. W. Wassall; Sec. L. L. Davis, Cor. Sec Geo. J. Dennis.

*Chicago Dental Club*—Meets on the fourth Monday of each month. Pres. A. B. Freeman; Sec. C. Stoddard Smith.

*Cleveland Dental Society.*—Meets the first Monday of each month. Pres. S. B. Dewey; Sec. Martha J. Robinson.

*Connecticut Valley Dental Society*—Meets annually. Next meeting at Hartford, May, 1894. Pres. H. Rider, Danbury, Mass.; Sec. Geo. A. Maxfield, Holyoke, Mass.

*Detroit Dental Society*—Meets monthly, Pres. E. C. Moore, Sec. H. C. Raymond.

*Eighth District Dental Society, N. Y.*—Next annual meeting April 24th, 1894, in Buffalo. Pres. F. J. Burkhart, Batavia; Sec., W. V. Grove, Buffalo.

*East Tennessee Dental Association*—Meets annually. Next meeting will be held in 1894, exact time and place not yet decided. Pres. U. D. Billmeyer, Chattanooga; Sec. and Treas. A. W. Palmer, Chattanooga.

*Fifth District Dental Society of N. Y.*—Meets semi-annually. Next annnal meeting will be held i Utica, April 9 and 10, 1894. Pres. S. E. McDougal, Clinton; Cor. Sec. A. R. Cooke, Syracuse.

*First District Dental Society of the State of New York*—Meets annually. The next meeting will be held on the second Tuesday of October, 1892. Pres., Wm. Carr, New York City; Sec., Benj. C. Nash, New York City.

*First District Dental Society of Illinois*—Next meeting will be held in Peoria, second Tuesday in September, 1894. Pres. O. M. Daymunde, of Monmouth; Sec. W. O. Butter, La Harpe.

*Knoxville City Dental Society*—Meets monthly. President, J. T. Cazier; Sec. A. J. Cottrell.

*Mississippi Valley Dental Society*—Meets annually at Cincinnati, next Meeting March, 1894. Pres., O. N. Heise, Cincinnati; Sec. H. T. Smith, Cincinnati; Cor. Sec. H. C. Matlack, Cincinnati, O.

*New England Dental Society*—Meets annually. Next meeting first Thursday in Oct., 1892, in Boston Mass. Pres. J. F. Adams, Worcester, N. H.; Sec. E. O. Kinsman, Cambridge, Mass.

*Northern Illinois Dental Society* —Meets annually. Next meeting at Aurora, in October, 1894. Pres. E. R. Warner, Chicago; Sec. J. W. Cormany, Mt. Carroll.

*Northern Ohio Dental Association*—Meets annually. Next meeting at Put-in-Bay, O., on the 2d Tuesday in May, 1894. Pres. S. B. Dewey, Cleveland; Rec. Sec. F. H. Knowlton, Akron; Cor. Sec. J. F. Dougherty, Canton.

*Northwestern Dental Association* (embraces portions of Dakota, Minnesota and Manitoba) meets annually. Next meeting at Detroit, Dak. on the fourth Tuesday in July, 1892. Pres. J. W. Cloes, Jamestown, Dak. Sec. S. J. Hill, Fargo, Dak.

*Odontological Society of Pennsylvania*—Meets on the first Saturday evening of each month, at 8 o'clock p. m., at 13th and Arch sts; Pres. Jas. Truman, Rec. sec. A W. Deane.

*Odontological Society of New York City*—Meets the third Tuesday of each month. Pres. Wm. Jarvie; Rec. Sec. E. T. Payne.

*Odontological Society of Cincinnati*—Meets monthly. Pres. G. Molyneaux; Sec. H. C. Matlack.

*Odontological Society of Chicago*—Meets monthly. Pres. W. V. B. Ames; Sec., and Treas., Louis Ottofy.

*Pennsylvania Association of Dental Surgeons*—Meets second Tuesday of each month. Pres. H. E. Roberts; Cor. Sec. Theo. F. Chupein.

*Pittsburg Dental Society*—Meets the last Tuesday Evening of each month. Pres. J. B. Williams, Homestead; Sec. N. L. Reinecke.

*San Francisco Dental Society*—Meets the second Monday evening of each month. Pres. W. A. Knowles; Sec. Chas. Morffew; Treas. J. J. Birge.

*Sixth District Dental Society of N. Y.*—Meets semi-annually. The annual meeting will be held the first week in May, 1894. Pres., Chas. W. McCall, Binghamton; Sec., T. B. Fuller, Binghamton.

*Seventh District Dental Society of Ohio*—Meets annually. Next meeting in May, 1894. Pres., O. N. Heise, Cincinnati; Sec., J. R. Callahan, Cincinnati.

*Southern Illinois Dental Society*- Meets annually. Next annual meeting will be held at Nashville, Ill., on the 3d Tuesday in October, 1894. Pres., L. B. Torrence, Chester; Sec., G. W. Entsminger, Carbondale.

*Southern Minnesota Dental Society*—Next meeting in Mankato, second Tuesday in April, 1894. Pres. M. B. Wood, Mankato; Sec. F. B. Kremer, Minneapolis.

*St. Louis Dental Society*—Meets first and third Tuesday in each month. Pres. Dr. DeCourcy Lindsley; Cor. Sec. Wm. Conrad; Rec. Sec. J. G. Pfaff.

*The Isaac Knapp Dental Coterie of Fort Wayne, Ind.*—Meets first and third Tuesday in each month. Pres., H. C. Sites; Sec., J. S. McCurdy.

*The Minneapolis Dental Society Monthly*—Pres. E. F. Clark, Sec. E. J. Morrison.

*The Tacoma Dental Society*—Meets monthly. Pres. M. C. Burns; Sec. W. E. Burkhart.

*Washington City Dental Society*—Meets 3d Tuesday of each month. Pres. J. H. P. Benson; Rec. Sec. J. Roland Walton.

### CONTENTS.

COMMUNICATIONS.

### THE DENTAL REGISTER,

**A MONTHLY MAGAZINE DEVOTED TO THE INTERESTS OF THE**

### DENTAL PROFESSION.

*Terms Reduced to* $2.00 *Per Year. Single Copies,* 25 *Cents.*

**EDITED BY PROF. J. TAFT, D.D.S.**

—AND—

**Published by SAM'L A. CROCKER & CO., Ohio Dental and Surgical Depot.**

The volume commences in January and closes in December.

The REGISTER being issued monthly is always fresh, therefore Indispensable to the practitioner who desires to keep posted up with the new inventions and improvements which are daily developed. Neither expense nor effort will be spared to give value and variety to its contents. Articles will be illustrated with well executed engravings whenever they will add to the interest or assist to explanation.

Its extensive circulation and frequency of issue make it one of the BEST DENTAL ADVERTISING MEDIUMS in the United States.

All subscriptions must begin with January or July.

Local or special notices, five lines or less, will be inserted for $3 each insertion; over five lines, 50 cts. per line.

All advertising bills payable in advance, or at furthest, after the issue of the first number containing the advertisement. All transient advertisements to be paid for in advance.

☞CONTRIBUTIONS SOLICITED.

Exclusively Editorial Communications should be addressed to the Editor.

The latest number of the REGISTER may be ordered with or without other good at any time, and all letters should be addressed to

